# A study on the nutritional status and body composition of children with Hutchinson–Gilford progeria syndrome

**DOI:** 10.1186/s13023-025-04164-7

**Published:** 2026-01-12

**Authors:** Qinmei Yu, Jingjing Wang, Haidong Fu, Jianhua Mao

**Affiliations:** Department of Nephrology, Children’s Hospital of Zhejiang University School of Medicine, National Clinical Research Center of Child Health, Hangzhou, China

**Keywords:** HGPS, Nutritional status, Body composition, Cardiovascular risks

## Abstract

**Objective:**

Hutchinson-Gilford Progeria Syndrome (HGPS) is a rare genetic disorder characterized by premature aging, severe growth retardation, and metabolic abnormalities. This study aimed to evaluate the growth, nutritional status, and body composition of HGPS patients, with a focus on fat and muscle distribution, to provide insights into potential nutritional and therapeutic interventions.

**Methods:**

Eight HGPS patients (aged ≥ 3 years) and 18 age- and sex-matched healthy controls were enrolled. Physical assessments, dietary surveys, and laboratory tests were conducted, including dual-energy X-ray absorptiometry (DXA) to analyze bone density, fat distribution, and muscle mass. Genetic testing confirmed *LMNA* mutations in all patients. Data on growth parameters, dietary intake, and metabolic profiles were collected and compared with controls.

**Results:**

HGPS patients exhibited severe growth retardation, with significant declines in height and weight by two months of age compared to controls. Dietary surveys revealed that 4 out of 8 subjects had sufficient energy intake, the energy intake was 91 ± 39% of the estimated basal metabolic rate. DXA analysis showed reduced bone density (Z-score: -2.82 ± 1.46), abnormal fat distribution (increased visceral fat and decreased subcutaneous fat, the T/L fat ratio and A/G fat ratio > 95th percentile for boys and girls in China), and significant reductions in total and limb muscle mass(< mean-2SD).

**Conclusion:**

HGPS patients experience profound growth retardation, metabolic dysfunction, and abnormal body composition, including reduced muscle mass and altered fat distribution. A nutrient-dense diet with increased protein intake, healthy fat intake, and supplementation with vitamin D, calcium, and zinc are essential to support growth and muscle maintenance. Tailored and individualised dietary interventions may further improve outcomes. Continuous monitoring and further research are needed to optimize interventions and enhance the quality of life for HGPS patients.

## Background

Hutchinson‒Gilford progeria syndrome (HGPS) is an extremely rare syndrome leading to premature death [1]. The Progeria Research Foundation (PRF) calculates the point prevalence of HGPS at 1 in 18 million to 1 in 20 million living individuals, with an estimated worldwide case number of 350–400 [2]. HGPS is caused by mutations in exon 11 or intron 11 in the lamin A/C (*LMNA*) gene. These include both classical (*c.1824 C > T*, *G608G*) and nonclassical mutations (other mutational sites), leading to the production of progeria proteins. The accumulation of these proteins can lead to nuclear aberrations [3] and affect the recruitment of transcription factors, epigenetic regulatory factors, and DNA repair enzymes, influencing both gene expression and DNA repair [4]. The most prominent clinical feature of HGPS is the premature and rapid onset of natural aging. Patients generally develop symptoms within one year after birth [5] that severely affect their development, including skin scleroderma, hair loss from the edge of the occipital region to the center of the head, progressive joint contracture, bone dysplasia, nail malnutrition, and abnormal tooth development, as well as the gradual development of conductive hearing loss, insulin resistance, and lipid metabolism disorders [6]. Patients with classic HGPS have an average lifespan of only 14.5 years, with cardiovascular and cerebrovascular diseases being the main causes of death [7]. HGPS patients typically suffer from severe growth retardation and generalized fatty malnutrition, yet there is a lack of in-depth analysis of their body composition. While pharmacological interventions, such as farnesyltransferase inhibitors (FTIs), have shown promise in alleviating some symptoms, comprehensive dietary strategies and nutritional interventions remain underexplored. Dual-energy X-ray absorptiometry (DXA) provides a direct and accurate measurement of bone density, body fat, and muscle mass [8]. This study aims to address these gaps by conducting physical assessments, dietary surveys, and laboratory tests on HGPS patients, with a particular focus on body composition analysis using dual-energy X-ray absorptiometry (DXA). By comparing these results with those of healthy children, we seek to establish a scientific foundation for a multidisciplinary approach to HGPS management. Differences in the regional distribution of fat and muscle tissues are associated with varying cardiovascular risks. Understanding the distribution of fat and muscle tissues is crucial for developing targeted treatment strategies, which may improve the quality of life and life expectancy of individuals with HGPS. While a few studies have examined total fat mass and lean body mass in HGPS patients [9, 10], the regional distribution of body fat and muscle has not been thoroughly analyzed. Through this study, we hope to contribute to the knowledge base of HGPS and provide a new theoretical foundation for potential nutritional and metabolic interventions that could help mitigate disease progression.

## Subjects and methods

### Subjects

The subjects enrolled in this study were HGPS patients who received treatment at the Children’s Hospital, Zhejiang University School of Medicine, from August 2020 to May 2023 and were aged 3 years and above (children under 3 years old cannot cooperate to undergo DXA detection). All the subjects were diagnosed through genetic confirmation. All patients were diagnosed with *LMNA* gene mutations through whole exome sequencing. The genetic diagnosis was performed early during the onset of the children’s illness. Additionally, we recruited **18** age- and sex-matched healthy children as the control group(unrelated personnel). The Medical Ethics Committee of the Children’s Hospital, Zhejiang University School of Medicine, approved this study (Approval No. 2021-IRBAL-108).

### Method

#### General information

The information of the patients hospitalized at the Children’s Hospital, Zhejiang University School of Medicine, as well as their general information, including sex, age, birth history (preterm birth, abnormal birth weight or height, mode of delivery, any abnormal reasons for cesarean section, and a history of asphyxia or resuscitation), height and weight, was collected.

#### Physical measurement and evaluation

Weight measurements were conducted via a digital scale to the nearest 0.1 kg and recorded in kilograms. A baby height board with an accuracy of 0.1 cm was used to measure the body length data. The patients with spinal curvature were summed using segmental height to obtain the final height. The consecutive height and weight data of the patients were retrospectively collected, with some data derived from annual physical examinations and others provided by the parents. We plotted the weight growth curve based on the WHO growth chart and compared it with children of the same age and sex.

#### Dietary survey

The 24-hour dietary survey method (retrospective method) was adopted. NutriStar software from Shanghai Zhen Ding Technology Co., Ltd., was used to record the food intake of children in the past 24 h and conduct analyses to calculate the actual energy intake of the children, as well as the percentages of carbohydrates, protein and fats in the total energy intake. NutriStar Software Recording Method: We input the child’s age, gender, weight, height, and daily dietary information, including the types of foods, quantities, and portion sizes. Based on this data, the software calculates whether the intake of each macronutrient and total energy aligns with the recommended nutritional standards, considering the child’s age, gender, and activity level. For instance, the recommended intake of energy, protein, fat, and carbohydrates varies across different age groups. Finally, NutriStar generates a detailed nutritional analysis report, displaying the intake of each macronutrient and its proportion relative to the recommended intake levels. We used the Schofield equation [11] (Females: Age < 3 years(16.252 W + 10.232 H-413.5); Age 3–10 years(16.969 W + 1.618 H + 371.2); Age 10–18 years(8.365 W + 4.65 H + 200.0). Males: Age < 3 years(0.167 W + 15.174 H-617.6); Age 3–10 years (19.59 W + 1.303 H + 414.9); Age 10–18 years (16.25 W + 1.372 H + 515.5); W = weight in kg; H = height in cm) to calculate each child’s energy requirements based on height and weight. If the actual intake was less than 80% of the basal metabolic rate, it was classified as insufficient energy or nutrient intake.

#### Laboratory examination

Hemoglobin; albumin; prealbumin; lipid metabolism including total cholesterol, triglycerides, High-Density Lipoprotein Cholesterol; bone metabolism including blood calcium, blood phosphorus, vitamin D, osteocalcin; insulin-like growth factor (IGF)-1 and binding protein 3; lactate dehydrogenase(U/L), alanine aminotransferase(U/L), creatine kinase(U/L), creatine kinase-MB activity(U/L); insulin resistance (fasting insulin (mU/L) × fasting blood glucose (mmol/L)/22.50 > 3 indicated insulin resistance) of the subjects were determined via laboratory examination(Roche Cobas c 311, Roche Cobas e411). Heart rate and blood pressure were measured; X-rays were used to detect the skull, chest, and pelvis plain films; anterior and lateral views of the ankle joint; and long bone films of the subjects (Philips Digital Diagnosis). the blood vessels in the neck and the heart were examined by b ultrasound (Philips EPIQ CVx). We analyzed subjects with joint deformities, joint dislocations, and osteolysis.

#### Measurement and analysis of body composition

The lumbar spine bone density, fat mass (e.g., fat mass index (FMI), total body fat percentage (BF%), trunk to leg (T/L) fat ratio and android to gynoid (A/G) fat ratio of the subjects were measured via dual-energy X-ray absorptiometry (DXA) (Hologic Discovery). The patient was supine on the scanning bed. The left foot and left leg were parallel to the scanning bed and rotated inward at 25° so that the lumbar spine and femur were in a vertical position, and the scan was carried out for 15 s. The muscle mass was measured on the basis of the muscle mass index (MMI), appendicular skeletal muscle mass index (ASMI), appendicular skeletal muscle mass ratio (ASMR), upper limb skeletal muscle mass index (ULSMI), and lower limb skeletal muscle mass index (LLSMI). The FMI, BF%, A/G fat ratio, T/L fat ratio, MMI, ASMI, ASMR, ULSMI, and LLSMI of the normal population matched by age and sex were used as references. BMDZ score<-2SD was defined as low bone density, The normal range for FMI, BF%, A/G fat ratio, and T/L fat ratio refers to the 5th-95th percentile range for children of the same age and sex in China. Values below the normal range are considered below the 5th percentile for children of the same age and sex in China, while values above the normal range are considered above the 95th percentile for children of the same age and sex in China [12]. The assessment of MMI, ASMI, ASMR, ULSMI, and LLSMI is typically based on age- and sex-matched reference databases. MMI, ASMI, ASMR, ULSMI, and LLSMI< -2SD is considered indicative of a reduction [13].

### Statistical analysis

Data processing and analysis were conducted via SPSS 26.0, and all the graphs were drawn via Origin 2021. The Shapiro-Wilk test is used to check for normality in the data. Continuous data conforming to a normal distribution are presented as the means ± standard deviations. Continuous data that did not conform to a normal distribution are represented by the median. The categorical variables are presented as absolute counts and percentages.

## Results

### General information

A total of eight HGPS patients (five males and three females) were enrolled, and all patients were diagnosed with HGPS by genetic tests. All patients had *LMNA* gene mutations, with *c.1824 C > T (exon 11) G608G* heterozygous mutations being the most common. Most of them had no abnormal birth history(preterm birth, birth asphyxia rescue history, etc.)and 2 patients had low birth weights. These patients developed symptoms shortly after birth. Moreover, the primary clinical manifestations are skin hardening and growth retardation. The genotype, onset age and primary manifestations of all the subjects are shown in Table 1.

**Table 1:** Genotypes and clinical characteristics of Chinese patients with HGPS M: male; F:female༛LMNA: lamin A/C gene༛Hom: Homozygous༛Het: heterozygous

*N*	Sex	Age of onset	Age	Initial symptoms	History of birth	Gene	GNE mutations	Protein	Hom/Het
1	M	1 month and 10 days	11 years and 10 months	Skin sclerosis , Growth retardation	Full term, natural delivery	*LMNA*	*c.1824 C > T (exon 11)*	*G608G*	Het
2	M	1 month	4 years and 10 months	Skin sclerosis	Full term, natural delivery	*LMNA*	*c.1824 C > T (exon 11)*	*G608G*	Het
3	M	1 month	9 years and 5 months	Skin sclerosis	Full term, cesarean section, low fetal movement	*LMNA*	*c.1824 C > T (exon 11)*	*G608G*	Het
4	F	3 months	7 years and 4 months	Skin sclerosis	Full term, natural delivery	*LMNA*	*c.1824 C > T (exon 11)*	*G608G*	Het
5	F	1 month − 3 months	10 years and 1 months	Skin sclerosis	Full term, cesarean section, low birth weight	*LMNA*	*c.1822G > A (exon 11)*	*G608S*	Het
6	F	6 years old	10 years and 8 months	Growth retardation	Full term, natural delivery	*LMNA*	*c.1968 + 5G > C (intron 11)*	*-*	Het
7	M	1 month ~ 3 months	4 years and 11 months	Skin sclerosis	Full term, natural delivery	*LMNA*	*c.1824 C > T (exon 11)*	*G608G*	Het
8	M	1 month ~ 3 months	11 years and 10 months	Skin sclerosis	Full term, natural delivery	*LMNA*	*c.1824 C > T (exon 11)*	*G608G*	Het

### Weights and heights

**Fig. 1:**
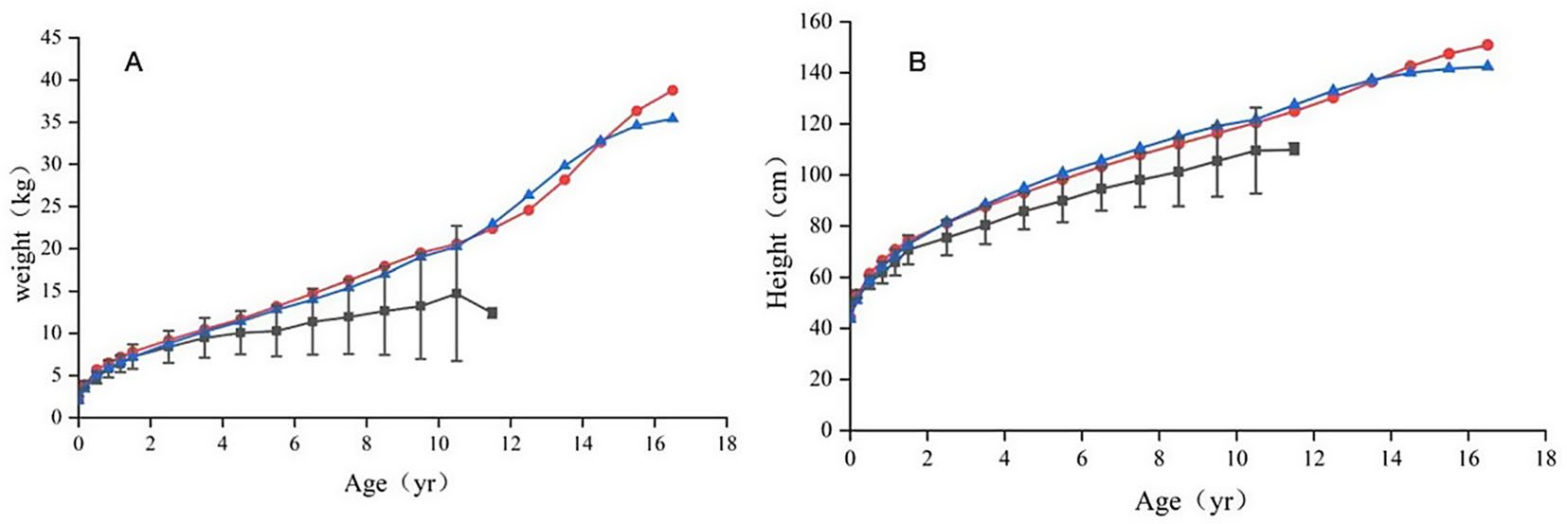
**A**, Weight plotted on the basis of age. The red lines represent the normal 3rd percentile for boys, and the blue lines represent the normal 3rd percentile for girls, which is approximately 2 standard deviations below the average. The black color represents the average weight (± SD) of all patients. **B**, The height plotted on the basis of age. The red lines represent the normal 3rd percentile for boys, and the blue lines represent the normal 3rd percentile for girls, which is approximately 2 standard deviations below the average. The black color represents the average height (± SD) of all patients. The point was centered around each time period. For example, all values between 0 and 4 months were averaged and plotted over 2 months

At birth, HGPS patients had an average weight of 2.93 ± 0.38 kg, with a weight-for-age Z-score of -0.64 ± 0.61, showing no significant difference compared to the healthy control group (3.21 ± 0.45, *p* > 0.05). Their average height was 49.86 ± 0.99 cm, with a height-for-age Z-score of 0.06 ± 0.93, also showing no significant difference compared to the control group (51.42 ± 2.89 cm, *p* > 0.05). However, by two months of age, the weight-for-age Z-score had declined to -2.83 ± 1.04, and the height-for-age Z-score to -2.45 ± 0.91, both of which were significantly lower than those of the healthy control group (*p* < 0.05). The weight and height of all the subjects were dropping below the 30th percentile at 2 months after birth. (Fig. 1; Table 2)

**Table 2:** Weight and height data calculated based on age points of Chinese patients with HGPS

Weight					Height				
HGPS			Healthy child		HGPS			Healthy child	
Age(m = monthy = years)	mean ± SD(kg)	Weight-for-Age Z-score	mean ± SD(kg)	p	Age(yr)	mean ± SD(cm)	Height-for-Age Z-score	mean ± SD(cm)	p
0	2.93 ± 0.38	-0.64 ± 0.61	3.21 ± 0.45	> 0.05	0	49.86 ± 0.99	0.06(0.93)	51.42 ± 2.89	> 0.05
2 m	3.93 ± 0.40	-2.83(1.04)	5.74 ± 0.51	< 0.05	0.167	53.38 ± 1.51	-2.45 ± 0.91	58.68 ± 3.41	< 0.05
6 m	4.74 ± 0.70	-3.078 ± 1.28	7.56 ± 1.38	< 0.05	0.5	58.00 ± 2.62	-4.30 ± 1.59	67.04 ± 4.29	< 0.05
10 m	5.79 ± 1.04	-3.74±-3.74	8.93 ± 2.05	< 0.05	0.833	61.88 ± 4.22	-4.80 ± 2.12	69.17 ± 5.08	< 0.05
1.167y	6.38 ± 1.00	-3.74 ± 1.37	10.01 ± 2.36	< 0.05	1.167	65.75 ± 5.01	-4.61 ± 2.19	77.28 ± 7.30	< 0.05
1.5y	7.23 ± 1.45	-3.43 ± 1.63	10.54 ± 2.76	< 0.05	1.5	70.63 ± 5.68	-4.03 ± 2.20	80.91 ± 7.81	< 0.05
2.5y	8.39 ± 1.91	-3.72 ± 1.73	12.84 ± 3.59	< 0.05	2.5	75.38 ± 6.91	-4.89 ± 2.13	90.62 ± 8.56	< 0.05
3.5y	9.46 ± 2.35	-3.79 ± 1.74	15.19 ± 4.78	< 0.05	3.5	80.25 ± 7.42	-5.01 ± 1.93	100.49 ± 10.45	< 0.05
4.5y	10.06 ± 2.57	-4.15 ± 1.70	17.56 ± 5.20	< 0.05	4.5	85.75 ± 7.11	-5.04 ± 1.63	105.91 ± 12.90	< 0.05
5.5y	10.27 ± 3.01	-4.70 ± 1.92	20.14 ± 5.92	< 0.05	5.5	89.86 ± 8.43	-4.68 ± 1.99	113.15 ± 13.67	< 0.05
6.5y	11.37 ± 3.91	-5.14(1.82)	22.59 ± 6.90	< 0.05	6.5	94.50 ± 8.55	-4.61 ± 1.67	120.39 ± 15.72	< 0.05
7.5y	11.95 ± 4.41	-5.38(1.99)	24.36 ± 7.01	< 0.05	7.5	98 ± 10.62	-4.72 ± 1.94	126.52 ± 15.93	< 0.05
8.5y	12.66 ± 5.24	-5.17 ± 2.19	28.42 ± 10.7	< 0.05	8.5	101.2 ± 13.50	-4.86 ± 2.30	130.10 ± 17.23	< 0.05
9.5y	13.22 ± 6.28	-5.37 ± 2.27	31.01 ± 12.83	< 0.05	9.5	105.4 ± 13.94	-4.82 ± 2.23	135.13 ± 16.82	< 0.05
10.5y	14.70 ± 8.00	-	34.33 ± 11.19	< 0.05	10.5	109.5 ± 16.82	-4.84 ± 2.57	141.51 ± 16.18	< 0.05
11.5y	12.40 ± 0.57	-	38.08 ± 15.91	< 0.05	11.5	109.8 ± 2.55	-5.29 ± 0.30	147.59 ± 18.25	< 0.05

### Dietary survey

A dietary survey was conducted on all patients, revealing that 4 out of 8 subjects had sufficient energy intake, while the remaining 4 had inadequate intake. The total energy intake was 91 ± 39% of the estimated basal metabolic rate. The macronutrient composition consisted of carbohydrates at 100 ± 54 g (2.39 ± 0.70 g/kg, 47 ± 3%), fats at 34 ± 13 g (39 ± 4%), proteins at 30 ± 17 g (14 ± 2%), Calcium(585.00 ± 76.53 mg/d), phosphorus(338.06 ± 32.37)mg/d, Magnesium(5.38 ± 0.42 mg/d). (Table 3)

**Table 3:** Analysis of the 24-hour retrospective dietary survey results of 8 children with HGPS

Intake types	Intake amounts	Proportion
The total energy intake(mean ± SD)	827 ± 401 kcal/d	91 ± 39.%(proportion of the basal metabolic rate)
Proteins(mean ± SD)	30 ± 17 g/d(2.39 ± 0.70 g/kg/d)	14 ± 2%(proportion of the total energy intake)
Fats(mean ± SD)	34 ± 13 g/d(2.81 ± 0.66 g/kg/d)	39 ± 4%(proportion of the total energy intake)
Carbohydrates(mean ± SD)	100 ± 54 g/d(7.87 ± 2.39 g/kg/d)	47 ± 3%(proportion of the total energy intake)
Calcium(mean ± SD)	585.00 ± 76.53 mg/d	8 out of 8 (8/8) below the normal range
phosphorus(mean ± SD)	338.06 ± 32.37 mg/d	7out of 8 (7/8) below the normal range
Magnesium(mean ± SD)	5.38 ± 0.42 mg/d	7out of 8 (7/8) below the normal range

### Laboratory and imaging examinations results

The average values of hemoglobin, albumin, and prealbumin were 136.91 ± 11.82 g/L, 43.71 ± 3.06 g/L, and 225.85 ± 51.03 mg/L, respectively. The blood calcium and phosphorus levels were 2.48 ± 0.09 mmol/L and 1.84 ± 0.28 mmol/L, respectively. The concentrations of vitamin D and osteocalcin were 73.68 ± 64.34 nmol and 26.78 ± 13.53 ng/mL, with decreases observed in 4 out of 8 patients (4/8) for vitamin D and 2 out of 8 patients (2/8) for osteocalcin. Regarding lipid metabolism, all patients (8/8) had normal total cholesterol (4.39 ± 4.31mmol/L), while 3 out of 8 patients (3/8) experienced an increase in triglycerides (2.30 ± 1.58mmol/L). A decrease in HDL-C was observed in 5 out of 8 patients (5/8) (1.03 ± 0.99mmol/L). Insulin resistance was present in 3 out of 8 patients (3/8). The average values of insulin-like growth factor (IGF)-1 and binding protein 3 were 143.18 ± 11.89 ng/mL and 4.76 ± 3.64 ug/ml, respectively. The average values of lactate dehydrogenase(U/L), alanine aminotransferase(U/L), creatine kinase(U/L), creatine kinase-MB activity(U/L) were 225.50 ± 31.67, 36.50(25.25, 50.00), 82.67 ± 42.44, 20.33 ± 7.10 respectively. In HGPS patients, the mean heart rate was 109 bpm (92, 135), mean systolic blood pressure was 120.88 ± 19.27 mmHg, and mean diastolic blood pressure was 72.13 ± 8.52 mmHg. Among the 8 patients: 2 had carotid atherosclerotic plaques, 2 showed carotid intima-media thickening, 5 exhibited cardiac dysfunction-specifically, 3 with right ventricular diastolic dysfunction and 2with biventricular dysfunction. In the skeletal system, joint deformity and osteolysis were present in all patients (8/8), while 3 out of 8 patients (3/8) had hip dislocation. No patients (0/8) had fractures. (Table 4)

**Table 4:** Laboratory and imaging examinations results of Chinese patients with HGPS

Variable	Study Group	Normal Range
Hemoglobin(g/L)	136.91 ± 11.82	110.00–155.00 g/L
Albumin(g/L)	43.71 ± 3.06	39.00–54.00 g/L
Prealbumin(mg/L)	225.85 ± 51.03	150.00-300.00
C-reactive protein(CRP mg/L)	5.23 ± 1.45	0-8 mg/L
Blood calcium(mmol/L)	2.48 ± 0.09	2.10-2.80mmol/L
Blood phosphorus(mmol/L)	1.84 ± 0.28	1.37-1.99mmol/L
Vitamin D(nmol)	73.68 ± 64.34	50.00-250.00mmol/L
Osteocalcin(ng/mL)	6.78 ± 13.53	12.70-97.60ng/ml
Total cholesterol(mmol/L)	4.39 ± 4.31	< 5.20mmol/L
Triglycerides(mmol/L)	2.30 ± 1.58	< 1.70mmol/L
HDL-C(mmol/L)	1.03 ± 0.99	> 1.15mmol/L
Insulin-like growth factor-1(ng/ml)	143.18 ± 11.89	18.00-172.00ng/ml
Binding protein 3(ug/ml)	4.76 ± 3.64	0.90-4.30ug/ml
Heart rate(bpm)	109(92, 135)	< 100
Systolic blood pressure, mmHg	120.88 ± 19.27	*P*5-*P*95
Diastolic pressure, mmHg	72.13 ± 8.52	*P*5-*P*95
Lactate dehydrogenase(U/L)	225.50 ± 31.67	110.00-295.00U/L
Alanine aminotransferase(U/L)	36.50(25.25, 50.00)	< 50.00U/L
Creatine kinase(U/L)	82.67 ± 42.44	39.00-308.00U/L
Creatine kinase-MB activity(U/L)	20.33 ± 7.10	< 24.00U/L
Carotid stenosis/plaque	4/8 (Carotid artery plaque: 2 cases; Carotid artery stenosis: 2 cases)	Normal
Cardiac insufficiency	5/8 (Right ventricular diastolic dysfunction: 3 cases; Biventricular dysfunction: 2 cases)	Normal
Joint deformity	8/8 (present in all patients)	Normal
Osteolysis	8/8 (present in all patients)	Normal
Hip dislocation	3/8 (3 cases presented with unilateral hip dislocation)	Normal
Fractures	0/8 (No patients had fractures)	Normal

### Examination by DXA

#### Bone density

**Fig. 2:**
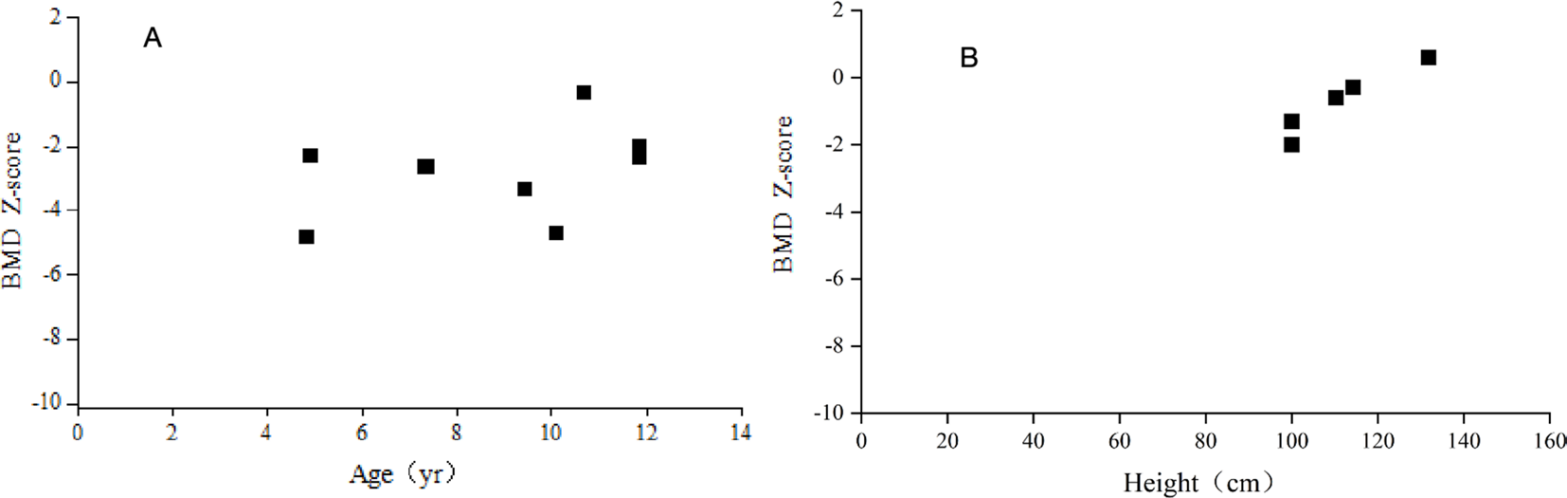
Bone mineral density Z score of 8 children with HGPS

The Z score detected by DXA was − 2.82 ± 1.46. Seven out of 8 patients had low bone density (Z score<-2). After the age was corrected for height, 5 patients aged 3 years and over were included, and the detected Z score was − 0.72 ± 0.99. (Fig. 2).

#### Total body fat percentage

**Fig. 3:**
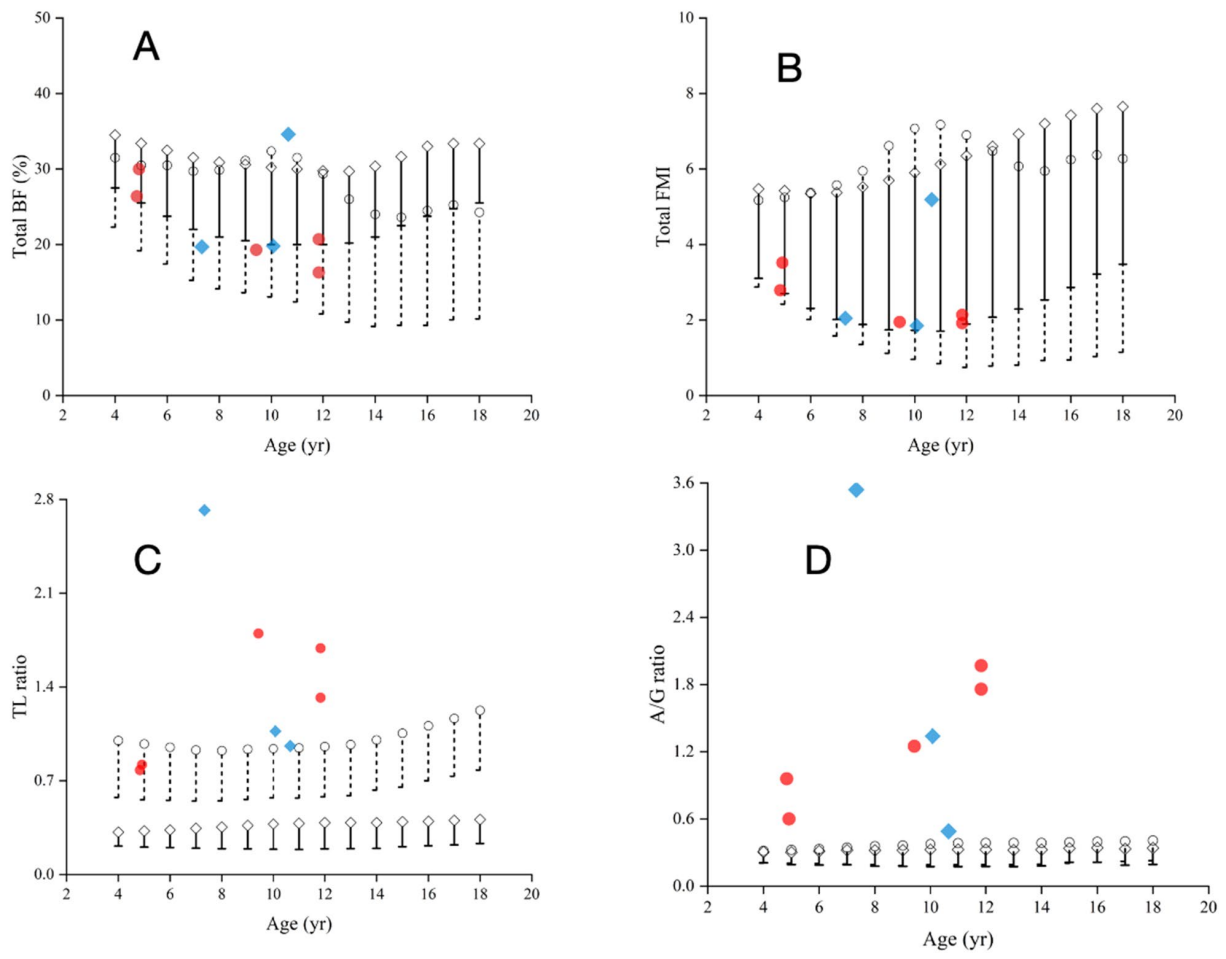
Total fat content and distribution of the subjects. **A**, the reference value of the FMI for age; **B**, the reference value of BF% for age; **C**, T/L fat ratio; **D**, male‒female A/G fat ratio. The bar represents the 5th–95th percentiles for boys and girls in China: the dotted bar represents boys, and the solid diamond represents girls. The red dots and blue diamonds refer to boys and girls, respectively

A total of 5 out of 8 (5/8) of HGPS patients had a total body fat percentage (BF%) within the normal range, 1 out of 8 (1/8) above the normal range (> 95th percentile for boys and girls in China), and 2 out of 8 (2/8) below the normal range (< 5th percentile for boys and girls in China) (Fig. 3A). The fat mass index (FMI) of all HGPS patients (8/8) was within the normal range (5th–95th percentile for boys and girls in China) (Fig. 3B). A total of 6 out of 8 (6/8) HGPS patients had a T/L fat ratio higher than the normal range (5th–95th percentile for boys and girls in China), whereas 2 out of 8 (2/8) had a T/L fat ratio within the normal range (Fig. 3C). All HGPS patients(8/8) had a male‒female A/G fat ratio higher than the normal range (5th–95th percentile for boys and girls in China). The results indicated that the T/L fat ratio and A/G fat ratio of most HGPS patients were greater than those of normal controls (5th–95th percentile for boys and girls in China) (Fig. 3D).

#### Skeletal muscle mass

**Fig. 4:**
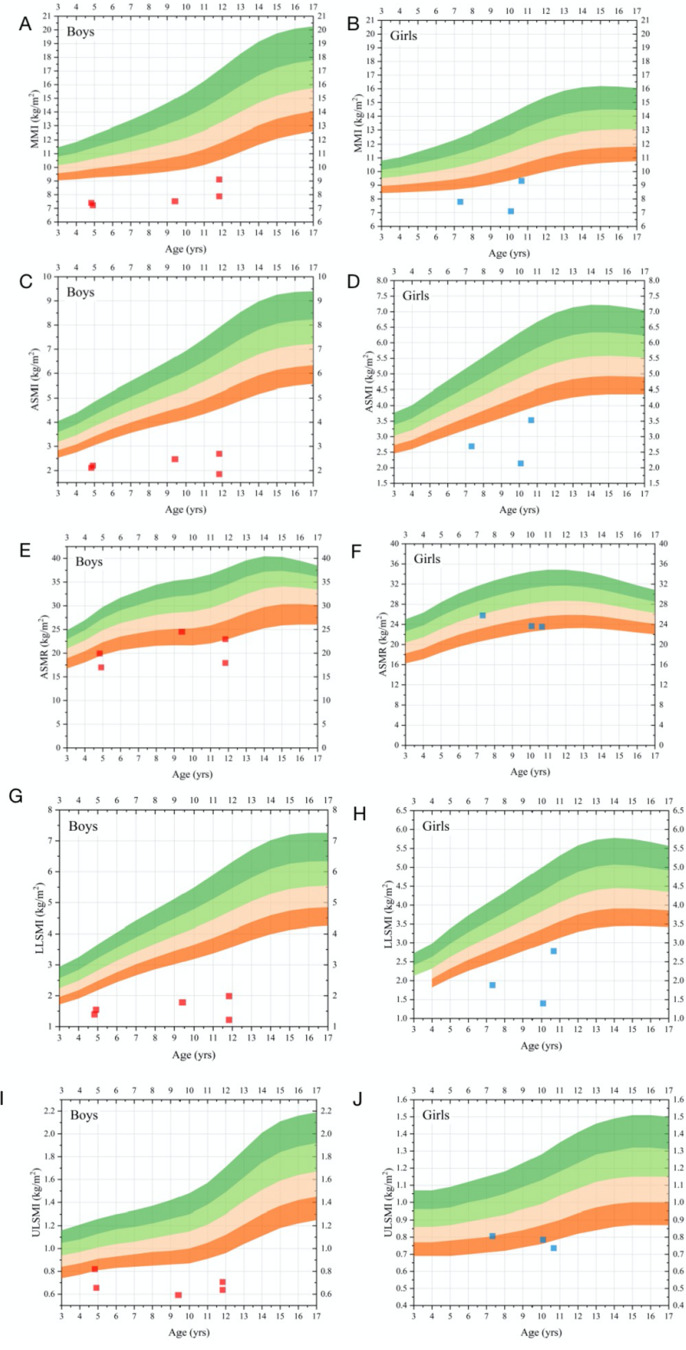
Reference curves of MMI (**A + B**), ASMI (**C + D**), ASMR (**E + F**), ULSMI (**G + H**), and LLSMI (**I + J**). Red area = mean-2SD to mean-SD; pink area = mean-SD to mean; cyan area = mean to mean + SD; green area = mean + SD to mean + 2SD. The red dots and blue diamonds refer to boys and girls, respectively. ASMI = appendicular skeletal muscle mass index; ASMR = appendicular skeletal muscle mass mass ratio; MMI = muscle mass index; LLSMI = lower limb skeletal muscle mass index; ULSMI = upper limb skeletal muscle mass index; SD = standard deviation

Examination via DXA revealed that (Fig. 4) the MMI of all the subjects was < mean-2SD. The ASMI of all the subjects was < mean-2SD. The ASMR of two subjects was < mean-2SD, the ASMR of five subjects ranged from the mean-SD to the mean-2SD, and the ASMR of one subject ranged from the mean-SD to the mean. The LLSMI of all the subjects was < the mean-2SD. The ULSMI of five subjects was < mean-2SD, the ULSMI of two subjects ranged from the mean-SD to the mean-2SD, and the ULSMI of one subject ranged from the mean-SD to the mean. The results indicated that there was a significant decrease in the total and appendicular muscle masses in most HGPS patients.

## Discussion

Hutchinson-Gilford Progeria Syndrome (HGPS) is a rare genetic disorder characterized by accelerated aging and various metabolic abnormalities. The main medical problem in HGPS is severe cardio- vascular disease, including generalized atherosclerosis and vascular calcification and stiffness, which ultimately provoke myocardial infarction, stroke, or heart failure, in this study, carotid artery abnormalities were detected in 4 patients—2 cases presented with carotid artery thickening and 2 with atherosclerotic plaques. Additionally, 5 children were found to have cardiac dysfunction. Therefore, regular assessments of growth and nutritional status, particularly the distribution of fat and muscle tissue, are essential for HGPS patients, as these factors are closely linked to the risk of cardiovascular disease [7, 14]. This study represents the first systematic investigation into the regional distribution of fat and muscle in HGPS patients, shedding light on their growth and development, skeletal health, and metabolic characteristics. This study provides the first comprehensive evaluation of growth patterns, dietary intake, and nutritional status in children with Hutchinson-Gilford Progeria Syndrome (HGPS), including pioneering systematic analysis of regional fat and muscle distribution. Our research findings provide insights for dietary guidance in children with progeria, establish an evidence-based foundation for personalized patient management of progeria, and point to new research directions for understanding this rare early-onset aging disease.

Children with HGPS show severe growth retardation. This study found that while patients’ average height and weight at birth were similar to those of healthy children, both measures showed a noticeable decline compared to healthy children by the age of two months. These findings are consistent with those of previous reports [15] and are related to the gene expression of progeria, heterochromatin organization, and failure to affect DNA repair [16]. However, the dietary survey in this study showed that the total energy intake was 91 ± 39% of the basal metabolic rate. Previous studies reported mean (± SE) energy intakes of 116 ± 8% based on predicted energy requirements and 125 ± 5% based on measurements of resting energy expenditure [10]. Overall, insufficient intake is unlikely to affect the children’s growth and development, except in cases of malocclusion or difficulty in mouth opening. Another study revealed that progerin has an inhibitory effect on growth and nutrition axes. Progerin accumulates outside the nucleus and gradually impairs the insulin-like growth factor 1 (IGF-1)/Akt signaling pathway by interacting with the IGF-1 receptor (IGF-1R) [17]. Treatment of HGPS patients with growth hormone can improve their growth and development. Abdenur JE, et al. [18] performed endocrine and metabolic tests on five HGPS patients, three of these patients received nutritional support and growth hormone treatment for 18, 6, and 12 months, respectively. The height of the patients increased to 5.00, 7.00, and 4.50 cm/year, accompanied by increased levels of IGF-I, IGF-II, and IGFBP-3, respectively. Ab Sadeghi-Nejad [19] reported one case of HGPS with low IGF-I. This patient responded normally to growth hormone stimulation, with an increased IGF-I level after growth hormone treatment. In this study, no abnormalities in the level of the insulin-like growth factor IGF-1 binding protein 3 were detected in children. However, no further growth hormone stimulation test was performed, and no growth hormone treatment was performed. The growth hormone status of children with HGPS thus requires further examination and analysis.

The skeletal system of children with HGPS exhibits progressive skeletal dysplasia [9]. Almost all children have varying degrees of joint deformity and osteolysis [2, 15], which was also verified in this study. Most children exhibit joint deformities and distal phalangeal dissolution, and osteolysis may also be present in the clavicle, mandible, ribs, and skull. It has been speculated that the accumulation of presenilin in the bone cells of HGPS patients causes toxicity and disordered cell signaling, ultimately leading to bone growth and cell death [9]. Additionally, due to progressive coxa valgus deformity and vascular dysfunction [20], HGPS patients are prone to joint dislocation [1], which may even be accompanied by joint ischemic necrosis. In this study, three patients had hip joint dislocation. Therefore, patients should not play on trampolines or other unstable surfaces to reduce the risk of hip joint dislocation.

Various studies have demonstrated that children with HGPS are susceptible to osteoporosis, as is the case in natural aging. In this study, the calcium and phosphorus levels of the patients were essentially normal, 50% of the patients presented with reduced levels of vitamin D. DXA revealed significant decreases in the bone densities of the patients. Therefore, children with HGPS should increase their intake of bone health-related nutrients together with regular supplementation with vitamin D to improve their bone development.

Nevertheless, unlike the overall demineralization observed in osteoporosis associated with natural aging [21], HGPS-induced osteoporosis results largely from focal and nonclassical demineralization, which does not significantly increase the risk of fracture [5]. Previous studies have demonstrated that fractures are not common in HGPS patients, and none of the 8 patients in this study developed any fractures. In addition, after correcting using the age and height, the DXA measurements revealed that the bone densities were within the normal range, indicating no increased risk of fracture in children with HGPS compared with healthy children of the same body type.

Fat is a highly active tissue that not only stores calories in the form of triglycerides but also secretes various compounds, including cytokines, chemokines, and hormone-like factors. Indeed, there are close physiological and pathological relationships between HGPS and fat formation. Mutations in lamin A in HGPS may impair the localization of the critical lipogenic transcription factor SREBP1c, adversely affecting fat formation [22]. Premature aging of cells may also be involved in the pathological process of adipo-dystrophy [23]. The present study revealed that despite significant reductions in subcutaneous fat in HGPS patients, their total blood cholesterol levels were normal, with varying degrees of elevated triglycerides and decreased high-density lipoprotein levels.

The regional distribution of body fat in children and adolescents is vital for understanding the etiology of cardiac metabolic risk. It has been reported that high fat deposition in the abdominal area plays an important role in the development and progression of cardiovascular disease, whereas a characteristic female fat distribution in the legs and buttocks is negatively correlated with the risk of cardiovascular metabolic diseases, suggesting that this type of fat distribution may protect against cardiac disorders [24, 25]. Compared with fat stored in the legs and buttocks, visceral fat is more resistant to insulin and secretes more proinflammatory cytokines, such as tumor necrosis factor-α or plasminogen activator inhibitors [26, 27]. In this study, the total fat percentage and total fat mass indices in the HGPS patients were mostly within the normal range, although there was a significant decrease in fat deposition in the legs and buttocks, which may increase the risk of cardiovascular.

Based on the above characteristics of abnormal lipid metabolism and distribution in children with progeria, relevant studies have clarified the distinct effects of foods and related factors on lipid metabolism: protective effects of foods include non-fat milk intake correlating with lower abdominal adiposity compared to high-fat milk [28], fibre and the ratio of whole grain to total grain showing the strongest inverse associations with adiposity indicators [29], daily intake of yellow pea flours (equivalent to half a cup of yellow peas) reducing insulin resistance (with whole pea flour (WPF) further lowering women’s android adiposity) [30], and tart cherry intake associating with less hyperlipidemia, fat mass, and abdominal fat [31]; Baru almonds—rich in protein, healthy fats, and antioxidants—have been studied for their effects on body composition and lipid markers in overweight/obese women [32]. In contrast, certain nutrients exert detrimental effects: fructose stimulates the expression of lipogenic genes to promote adipose fat deposition and abdominal adiposity [33], and it also activates inflammatory pathways in tissues, which suppresses insulin signaling and leads to systemic insulin resistance. Additionally, other factors influence lipid metabolism: uric acid improves abdominal adiposity, blood glucose, and lipid levels in mice through absorptive and metabolic targets, a higher dietary acid load increases the risk of abdominal obesity (with this effect being more pronounced in women) [34], and the MeDiet may counteract the harmful effects of increased adiposity on the risk of cardiovascular health [35]. These findings highlight how different food types and nutrients shape lipid metabolism, thereby offering evidence-based support for developing dietary plans that benefit lipid metabolism in children with progeria.

Muscles play a central role in systemic protein metabolism, serving as the leading providers of amino acids, maintaining protein synthesis in important tissues and organs, and providing liver glycogen precursors. Skeletal muscles are considered the principal sites for the processing of insulin-induced glucose, accounting for 85% of insulin-induced glucose utilization. Low skeletal muscle mass is associated with multiple metabolic risk factors [36]. High muscle mass can reduce the risk of osteoporosis and contribute to cardiovascular health [37]. Our results revealed a significant decrease in both total and limb muscle mass in most children with HGPS. Research indicates that protein, vitamin D, and a balanced diet play a role in mitigating the impacts of low muscle mass [38], while extra virgin olive oil, a core component of the Mediterranean Diet, contributes to maintaining muscle mass and preserving muscle function [39].

Children with Hutchinson-Gilford Progeria Syndrome (HGPS) often experience growth retardation, making it essential to provide them with high-calorie, nutrient-dense foods to meet their energy needs. The HGPS Research Foundation recommends offering these children nutrient-rich, high-calorie foods and supplements to support their overall health. A well-balanced intake of carbohydrates, fats, and proteins is crucial. Dietary surveys indicate that children with HGPS generally maintain a reasonable macronutrient distribution. However, our laboratory data reveal varying degrees of elevated triglycerides and decreased high-density lipoprotein (HDL) levels, along with an increased Android to Gynoid (A/G) fat ratio and T/L ratio, suggesting a tendency for abdominal fat accumulation. To reduce visceral fat, a low-carbohydrate diet is recommended, as it can effectively decrease abdominal fat deposition by using skimmed milk powder instead of high-fat milk powder, increasing healthy fats (e.g., olive oil, nuts), and enhancing high-quality protein intake (e.g., lean meat, fish, protein powder, and plant-based proteins like beans and nuts). A high-fiber diet further supports weight management and fat distribution by improving gut health, stabilizing blood sugar and insulin levels, and reducing visceral fat accumulation, with key sources of dietary fiber including whole grains, vegetables, fruits, and legumes.

While diet plays a crucial role in body composition, resistance training is also highly beneficial, as regular physical activity enhances muscle metabolism, promotes lean muscle growth, and helps reduce overall fat accumulation. Therefore, HGPS children should engage in safe and appropriate physical activities to support their musculoskeletal health. Vitamin D is essential for bone health and may also influence the progression of HGPS, as research suggests that vitamin D receptor signaling can improve HGPS-related cellular dysfunctions, including DNA repair defects and premature aging. Supplementing with vitamin D may help alleviate some symptoms associated with the disease. Additionally, calcium and magnesium play a crucial role in muscle function and fat distribution. While our laboratory findings did not indicate deficiencies in these minerals, supplementation may help reduce muscle atrophy and promote healthier fat distribution.

Emerging studies suggest that high-calorie, high-fat diets may delay aging in HGPS mouse models [40], with findings indicating a near doubling of lifespan. However, dietary strategies should be tailored to each patient’s individual metabolic profile and specific needs. Nutritional interventions should be closely guided by healthcare professionals, including doctors and dietitians. Regular monitoring of a child’s growth and nutritional status is essential, and dietary adjustments should be made to ensure that nutrient intake meets the demands of growth, bone health, and muscle development.

However, this study has certain limitations in that it is a cross-sectional survey with a relatively small sample size, potentially leading to bias. Additionally, this study utilized the 24-hour dietary recall method, which offers operational simplicity but may be subject to recall bias and limited representativeness of habitual intake due to single-day assessment. Future research could incorporate multi-day dietary records or objective biomarkers to enhance data accuracy.

## Conclusion

Hutchinson-Gilford Progeria Syndrome (HGPS) is characterized by severe growth retardation, metabolic dysfunction, and abnormalities in fat and muscle distribution, leading to an increased risk of cardiovascular disease and skeletal disorders. This study highlights the necessity of regular assessments of growth, body composition, and nutritional intake in HGPS patients. Findings suggest that while overall energy intake may not be significantly deficient, impaired IGF-1 signaling and metabolic dysregulation contribute to growth failure. Additionally, HGPS patients exhibit reduced subcutaneous fat, abnormal fat distribution with increased visceral fat, and decreased muscle mass, all of which may exacerbate metabolic and cardiovascular risks. Nutritional interventions, including high-calorie, nutrient-dense diets, increased protein intake, and healthy fat consumption, are essential for supporting growth and muscle maintenance. Low-carbohydrate and high-fiber diets may help manage visceral fat accumulation, while vitamin D, calcium, and magnesium supplementation can promote bone and muscle health. Resistance training and carefully monitored physical activity may further aid in maintaining muscle mass and reducing metabolic risks. Given the progressive nature of HGPS, continuous monitoring and individualized nutritional and medical strategies are crucial. Emerging research on dietary modifications and targeted therapies offers potential avenues for improving patient outcomes. However, further longitudinal studies with larger sample sizes are needed to validate these findings and optimize intervention strategies for HGPS patients.

## Data Availability

The datasets used and/or analyzed during the current study are available from the corresponding author on reasonable request.
